# Intrapartum cardiotocographic patterns and perinatal outcomes in extremely preterm births: an exploratory retrospective cohort study

**DOI:** 10.1186/s12884-026-08954-0

**Published:** 2026-03-27

**Authors:** Nadine D de Klerk, Ivar R de Vries, Jeanne P Dieleman, Annemarie F Fransen, S Guid Oei

**Affiliations:** 1 Department of Gynecology and Obstetrics, Máxima MC, De Run 4600, Veldhoven, 5504 DB The Netherlands; 2 Department of Electrical Engineering, Eindhoven University of Technology, P.O. Box 513, Eindhoven, 5600 MB The Netherlands; 3 Department of Science, Máxima MC, De Run 4600, Veldhoven, 5504 DB The Netherlands; 4 Department of Gynecology and Obstetrics, Zuyderland Medical Center, Henri Dunantstraat 5, Heerlen, 6419 PC The Netherlands

**Keywords:** Extremely preterm birth, Perinatal outcome, Premature, Cardiotocography, Fetal monitoring, Intrapartum, Cohort

## Abstract

**Background:**

Extremely preterm birth (24–28 weeks of gestation) carries high perinatal risk. While intrapartum cardiotocography (CTG) is routinely used at term, its interpretation in extremely preterm birth remains unclear. This study aims to explore the relationship between intrapartum CTG characteristics and perinatal outcomes in extremely preterm birth.

**Methods:**

An exploratory retrospective cohort study, part of the NIEM-O study, was conducted at the Obstetrics Department of a tertiary referral hospital. Intrapartum CTG recordings of 73 women with a spontaneous onset of birth between 24 and 28 weeks of gestation were included. Signal loss (*n* = 73) and CTG characteristics during the last hour of the first stage of labor (*n* = 55), and during second stage of labor (*n* = 24) were assessed using computerized analysis. CTG characteristics (baseline fetal heart rate, heart rate variability, accelerations, decelerations, uterine contractions, and combinations of these characteristics) were compared between fetuses with neonatal outcome of low 5-minute Apgar score (< 7) and a group with 5-minute Apgar score > 7.

**Results:**

CTG tracings in preterm fetuses frequently displayed recognizable features, including baseline variability (median 18 bpm) and both accelerative (median five/hour) and decelerative (median 11/hour) responses. Preterm fetuses with a low 5-minute Apgar score (*n* = 20) exhibited significantly more variable decelerations (median 14/hour vs. six/hour) and uterine contractions (19/hour vs. 13/hour), compared to those without this outcome. CTG characteristics commonly described as indicators of hypoxia in term fetuses were not consistently observed in the studied preterm population.

**Conclusions:**

Although CTG features were often identifiable in preterm fetuses, their presentation differed from those commonly described at term, particularly in those with low 5-minute Apgar score. These findings highlight the need for gestational age-specific CTG reference ranges. Adapting CTG interpretation to reflect the unique physiology of this group is essential for improving timely clinical decision-making and reducing perinatal morbidity and mortality.

**Trial registration:**

Registered on 22 November 2023 in Clinicaltrails.gov (Number: NCT06151613) via https://clinicaltrials.gov/study/NCT06151613 and on 18 October 2022 to the Central Committee on Research Involving Human Subjects (NL82869.015.22).

**Supplementary Information:**

The online version contains supplementary material available at 10.1186/s12884-026-08954-0.

## Background

Cardiotocography (CTG) monitors fetal heart rate (FHR), maternal heart rate, and uterine activity, and is standard care in high-risk pregnancies, including those complicated by preeclampsia, fetal growth restriction, or imminent preterm birth [1, 2]. It remains the only accepted intrapartum monitoring tool for preterm fetuses [1]. During labor, changes in CTG patterns may be indicative of the fetus’ pathophysiological responses to fetal hypoxia and related perinatal asphyxia [3, 4]. Recognition of these patterns allows for prompt intervention, which may avert severe perinatal outcomes [1, 4].

In term pregnancies, the association between CTG features, such as baseline FHR, variability, accelerations, and decelerations, and perinatal outcomes is well-described in the literature [1, 4]. A comprehensive understanding of fetal physiology during labor is crucial for the accurate interpretation and appropriate management of CTG patterns, which may, in turn, affect perinatal outcomes [4, 5]. Nevertheless, it is often difficult to predict the significance of abnormal CTG patterns, leading to over and under-intervention in clinical practice [6–9]. Furthermore, loss of the CTG signal is a frequently encountered problem during labor, and only the FIGO guidelines present a definition and quantification of adequate single quality (up to 20% signal loss is deemed acceptable) [1]. Additionally, a recent study found a threshold of > 30% FHR signal dropout was found to be independently associated with asphyxia [10].

Notably, it is unclear whether CTG data from term pregnancies can be extrapolated to preterm fetuses, given their distinct physiology and increased susceptibility to hypoxia and acidemia [11, 12]. However, in clinical practice, CTG guidelines developed largely on term data, are commonly used for preterm fetuses [1, 13–15]. This is caused by a lack of knowledge and no existing CTG guidelines for preterm fetuses.

Even less is known about CTG tracings in the group of extremely preterm fetuses (gestational age < 28 weeks) during labor. Some studies report high rates of non-reassuring CTG patterns without increased mortality [16], while others find a much higher incidence of hypoxic injury in preterm infants with abnormal tracings (70–80% vs. 20% in term infants) [17–19].

Additionally, signal quality of intrapartum monitoring specifically in preterm fetuses has been investigated in only one study and showed a mean signal loss of 13% during the first stage of labor and 30% during the second stage [20]. To broaden our limited understanding of preterm intrapartum monitoring, this study aims to further explore the relationship between intrapartum CTG characteristics and perinatal outcomes, as well as the quality of the registration, in extremely preterm pregnancies (24–28 weeks’ gestation).

## Methods

### Population and setting

We conducted an exploratory retrospective cohort study involving women with a spontaneous onset of labor in Máxima MC, a tertiary teaching hospital with a neonatal intensive care unit in the Netherlands. Women giving birth between 24 + 0 and 28 + 0 weeks of gestation from 2014 to 2019, and with a minimum of 60 min CTG registration before birth, were included. This study was part of the NIEM-O study [21] (NCT06151613), which was approved by the Medical Ethics Committee of Máxima MC (NL82869.015.22). This committee waived the requirement for informed consent for the retrospective cohort, based on the study’s scale and a proportionality assessment of research burden relative to patient burden, in accordance with General Data Protection Regulation act.

### Data collection

All data pertaining to the pregnancy, labor, delivery, and neonatal period, as well as the CTG tracings, were retrieved from the hospital’s computerized patient records (Hix version 6.2). This was done by medically trained researchers using CTcue (v4.15.2), a platform for querying electronic health records, completed manually using structured data collection forms. All CTG tracings were recorded using the FM30 fetal monitor (Philips Healthcare). The Philips FM30 provides raw FHR and uterine activity signals with automated cross‑channel verification, but does not perform computerized CTG‑feature detection. CTG tracings covering the hour prior to expulsion (last hour of first stage of labor) and expulsion period (second stage of labor) were collected as two separate timeframes. In case of secondary caesarean section (CS), the hour before the intervention was taken as last hour of first stage, and the second stage was considered not applicable. Signal loss was calculated and all CTG registrations exceeding 33% loss of FHR signal were excluded [22].

### Definition and classification of CTG characteristics and hypoxia patterns

CTG characteristics that were evaluated included FHR baseline, fetal heart rate variability (fHRV), accelerations, decelerations and uterine activity. A normal baseline FHR was defined as 110–160 beats per minute (bpm). Tachycardia was defined as a baseline FHR above 160 bpm lasting more than 10 min [1]. Normal fHRV was defined as an average bandwidth amplitude (i.e., long-term variability) of 5 − 25 bpm in 1-min segments. Reduced fHRV was defined as a bandwidth amplitude between 2 and 4 bpm and an absent fHRV as below 2 bpm [1]. Accelerations were described as an increase in heart rate from the baseline, with a minimum duration of 15 s and a minimum amplitude of 15 bpm [1, 23, 24]. Decelerations were described as a decrease in heart rate from the baseline, with a duration of at least 15 s and a minimum amplitude of 15 bpm [1, 23, 24]. Uncomplicated variable decelerations were defined as a maximum duration of 60 s [25] and complicated variable decelerations as a minimum amplitude of 60 bpm, a minimum duration of 90 s. and recovery phase at baseline within 60 s [4]. Other variable decelerations were classified as non-classifiable variable deceleration. Late decelerations had a gradual onset, a gradual return to the baseline and/or reduced variability within the deceleration. Gradual onset and return occurred when more than 30 s elapses between the beginning/end of a deceleration and its nadir [1]. The duration threshold for a contraction was 25 s [26].

Based on fetal pathophysiology, combinations of (changes in) CTG characteristics were classified into patterns suggestive of progressive compromise [4]:

- Patterns suggestive of slowly evolving hypoxia:
  1. Normal baseline and fHRV with decelerations;
  2. Tachycardia with decelerations and normal fHRV;
  3. Tachycardia with decelerations and reduced fHRV.
- Patterns suggestive of subacute hypoxia:
  1. Complicated variable decelerations with normal fHRV;
  2. Complicated variable decelerations with reduced fHRV.
- Patterns suggestive of acute hypoxia:
  - Baseline FHR <80 bpm and loss of fHRV

Individual CTG features were defined according to the FIGO guideline (2015) [1] to ensure comparability with existing literature. In addition, combinations of these features were grouped into patterns suggestive of slowly evolving, subacute, or acute compromise, based on pathophysiological explanatory models described in prior literature [4]. This hybrid approach strengthens our study by reflecting both the prevailing clinical standard and the underlying physiological mechanisms, thereby enhancing interpretability and clinical relevance. Patterns were classified into gradually evolving, subacute, and acute hypoxia, as these reflect intrapartum pathophysiological changes. Chronic hypoxia was not included because it is primarily an antepartum condition and cannot be reliably assessed from intrapartum CTG alone [27]. Since gestational age-specific reference values for preterm CTG characteristics are lacking, definitions used for term fetuses were applied, in line with current clinical practice [1].

As it is known that visual assessment of a CTG by different physicians and midwives exhibits interobserver and intraobserver variability, the research team has developed a standardized approach to assess variables within a CTG [28, 29]. An algorithm was developed, based on previous literature [26, 30]. This algorithm was executed using MATLAB and combines the algorithm for detection of baseline FHR along with accelerations and decelerations as developed by Pardey et al. [30] with the algorithm for contraction detection developed by Vlemminx et al. [26]. The algorithm processed the raw fetal heart rate and uterine activity signals to identify baseline, variability, accelerations, decelerations and uterine contractions according to predefined duration and amplitude criteria derived from established guidelines. Combinations of detected features were subsequently grouped into predefined pathophysiological CTG pattern categories. This structure ensured consistency and reproducibility in feature extraction. Subsequently, a random sample (10%) of the CTG outputs was manually assessed to evaluate the algorithm’s accuracy and reliability. This approach reduced the known inter- and intra-observer variability in visual interpretation and increased reproducibility of the analysis [9].

### Outcomes

The study was designed to assess whether specific CTG features observed during labor are associated with adverse neonatal outcomes, thereby evaluating the predictive value of intrapartum monitoring in extremely preterm deliveries. The primary outcome was defined as low 5-minute Apgar score (< 7). This measure was selected because the Apgar score remains a clinically relevant and pragmatic indicator of neonatal condition immediately after birth [31]. Although the Apgar score does not directly quantify acid–base status, it reflects the infant’s overall physiologic adaptation and response to resuscitation efforts [31]. Importantly, robust evidence demonstrates that the 5-minute Apgar score has strong prognostic value for neonatal survival and severe morbidity, even among very preterm infants (< 32 weeks’ gestation) [32, 33]. This outcome was also chosen for practical reasons: umbilical cord blood gas values, while informative, are frequently missing in very preterm or emergency deliveries due to technical challenges and clinical priorities during resuscitation. Missingness is often non-random and more common in compromised neonates, which can introduce bias in population-based studies. In contrast, Apgar scores are assigned to all newborns and are almost universally recorded, even during resuscitation, making them a consistently available measure of neonatal condition in both routine and high-acuity settings [31]. These considerations support the use of the 5-minute Apgar score as a reliable and clinically meaningful primary outcome in this context. Nevertheless, biochemical evidence of asphyxia defined as (pH < 7.0 and base deficit ≥ 12mmol/L) was included as secondary outcome [34]. Further secondary outcome was defined as a composite outcome of perinatal mortality (deaths occurring after ≥ 22 weeks of gestation or, if unknown, birthweight > 500 gram and before 28 days postnatal [35]) and/or severe perinatal morbidity, defined as either intraventricular hemorrhage (IVH) grade three or more, periventricular leukomalacia (PVL) grade two or more, moderate or severe bronchopulmonary dysplasia (BPD), necrotizing enterocolitis (NEC) grade two or more or retinopathy of prematurity (ROP) necessitating laser therapy.

### Analysis

SPSS statistics (version 29.0, IBM) was used for the statistical analysis. Descriptive statistics were used as appropriate to summarize patient characteristics and CTG characteristics. To assess potential selection bias, a sensitivity analysis was performed comparing the incidence of the primary outcome between excluded and included patients. For comparisons between patients with and without the concerned outcome of interest, the unpaired t-test was used for normally distributed continuous data, the Mann Whitney U test for non-normally distributed continuous data and the Chi-square tests (χ²), with bootstrapping was applied in case of small cell counts for categorical data. Logistic regression analysis was performed to explore the relationship between the (combination of) CTG characteristics and the different outcomes, while accounting for potential confounders. Identified confounders for the primary outcome were Nullipara and corticosteroids (CCS). The restricted sample size precluded the incorporation of further confounding variables, despite their clinical relevance and support in existing literature, to avoid model overfitting and preserve statistical power. Inherent to the study design, there were no missing data for CTG characteristics, the primary outcome, or the predefined confounders. Umbilical cord blood gas values, used to define biochemical asphyxia, were available in 51 of 55 cases, resulting in four missing values for this secondary outcome. Two-sided p-values below 0.05 were considered statistically significant.

## Results

Between 2014 and 2019, 171 pregnant women were admitted to the Obstetric High Care and delivered extremely preterm. Of these women, 73 had a spontaneous onset of labor and had at least 60-minute CTG monitoring prior to birth. More than 33% signal loss was found in eighteen of the 73 CTGs (25%) in the first stage of labor, and in 36 of the 60 CTGs (60%) in the second stage of labor. The second stage of labor was absent in thirteen cases due to secondary caesarean section. No significant differences in outcome were observed between the included and excluded cases. Ultimately, 55 CTGs were included in the CTG characteristics analysis of the first stage, and 24 CTGs in the second stage analysis.

There were 20 (36%) cases of the primary outcome (low 5-minute Apgar score). Although baseline characteristics were generally similar between groups, cases with the outcome more frequently involved multiparous women, lower gestational age, female infants, epidural analgesia, and lower exposure to corticosteroids (Table 1).

Only CTG recordings with a continuous duration during the last 60 min of the first stage of labor were included. The median duration of CTG recording during the second stage was 11 min (IQR 2–23). Median signal loss was 15% (IQR 8–21) in the first stage and 20% (IQR 8–25) in the second stage.

**Table 1:** Baseline characteristics

	Total (*n* = 55)	Low Apgar score^a^ (*n* = 20)	Reference group^a^ (*n* = 35)	*P*-value
**Maternal characteristics**				
Ethnicity (n, %)				0.577^b^
Caucasian	46 (84%)	17 (85%)	29 (83%)	
Non-Caucasian	9 (16%)	3 (15%)	6 (17%)	
Age (Mean, SD)	29 (5)	30 (5)	30 (6)	0.768^c^
BMI, pre pregnancy (Mean, SD)	26 (5)	25 (6)	26 (4)	0.264^c^
Smoking (n, %)				0.696^b^
No smoking	48 (87%)	17 (85%)	31 (89%)	
Smoking	7 (13%)	3 (15%)	4 (11%)	
**Obstetrical characteristics**				
First Gestation (n, %)	30 (55%)	8 (40%)	22 (63%)	0.101^b^
Nullipara (n, %)	38 (69%)	**9 (45%)**	**29 (83%)**	0.003* ^**b**^
Gestational Age, in days (Mean, SD)	184 (8)	182 (9)	186 (7)	0.070^c^
Obstetrical disorder (n, %)				
Preeclampsia	0 (0%)	0 (0%)	0 (0%)	-^d^
Fetal growth restriction	3 (6%)	1 (5%)	2 (6%)	0.703^b^
Intra-amniotic infection	1 (2%)	0 (0%)	1 (3%)	0.636^b^
Vaginal blood loss	27 (49%)	9 (45%)	18 (51%)	0.646^b^
Maternal diabetes	3 (6%)	2 (10%)	1 (3%)	0.546^b^
PPROM (n, %)	28 (51%)	11 (55%)	17 (49%)	0.646^b^
Medication during or in short period before delivery (n, %)				
Tocolytics	50 (91%)	17 (85%)	33 (94%)	0.342^b^
Corticosteroids				0.092^b^
None	0 (0%)	0 (0%)	0 (0%)	
<2 days	16 (29%)	9 (45%)	7 (20%)	
2–14 days	35 (64%)	9 (45%)	26 (74%)	
>14 days	4 (7%)	2 (10%)	2 (6%)	
Magnesium sulfate	53 (96%)	19 (95%)	34 (97%)	1.000^b^
Epidural analgesia (n, %)	11 (20%)	6 (30%)	5 (14%)	0.181^b^
Caesarean section (n, %)	8 (15%)	2 (10%)	6 (17%)	0.696^b^
**Child characteristics**				
Male sex (n, %)	25 (46%)	6 (30%)	19 (54%)	0.082^b^

* Statistical significance (*p* < 0,05)

^a^ Low Apgar group: Apgar < 7 at 5 min; Reference group: Apgar ≥ 7 at 5 min

^b^ Chi-square test including bootstrapping, for categorical data

^c^Unpaired t-test, for normally continuous data

^d^ No analysis possible due to no cases

**Fig. 1:**
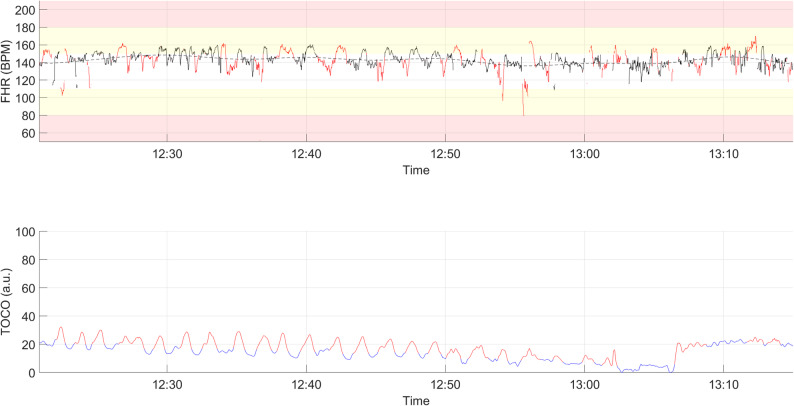
Example of a cardiotocographic trace during the last hour of the first stage of labor, with computerized classification of cardiotocographic features. Clinical context: 27 + 6 weeks’ gestation; persistent heavy vaginal bleeding and breech presentation in current pregnancy, leading to emergency secondary caesarean section under general anesthesia. The neonate was delivered in breech presentation with Apgar scores of 1/6/8 at 1/5/10 minutes. Postnatally, the infant developed necrotizing enterocolitis and pulmonary complications after which the infant died. The black line represents the measured fetal heart rate; black dashed line indicates the estimated fetal heart rate baseline; blue line shows the TOCO signal denoting uterine activity. Red markers denote detected events: decelerations, accelerations, or uterine contractions. FHR=fetal heart rate, bpm= beats per minute, a.u.= arbitrary units

Figure 1 presents a representative CTG trace from one of the study participants, with key CTG features annotated for illustrative purposes. The displayed CTG features are representative of the types of features commonly observed across the cohort, although the clinical scenario of this specific participant was atypical.

CTG analysis of all included participants in the last hour of the first stage of labor showed a median baseline FHR of 145 bpm, a median fHRV of 18 bpm, a median of five accelerations, eleven variable decelerations - of which ten were classified as uncomplicated variable decelerations and one as non-classifiable variable deceleration -, four late decelerations and sixteen uterine contractions (Table 2). The group with low Apgar score exhibited significantly more decelerations, particularly uncomplicated variable decelerations, compared to the reference group (i.e., those without low Apgar score). Additionally, more uterine contractions were observed in the group with low Apgar score compared to the reference group.

**Table 2:** Cardiotocographic (CTG) characteristics during the first and second stage of labor

CTG Characteristics (Median, IQR)^a^	First stage of labor						Second stage of labor									
	Total (*n* = 55)	Low Apgar score^b^ (*n* = 20)	Reference group^b^ (*n* = 35 )	*P*-value ^c^	OR (95% CI)	*P*-value ^d^	Total (*n* = 24)	Low Apgar score^b^ (*n* = 6)		Reference group^b^ (*n* = 18)	*P*-value^c^		OR (95% CI)		*P*-value^e^	
Baseline FHR, bpm	145 (139–151)	145 (139–153)	145 (139–150)	0.576	1.03 (0.96–1.11)	0.414	143 (137–148)	143 (135–146)	142 (137–150)			0.689		0.997 (0.927–1.073)		0.944
fHRV, bpm	18 (15–23)	18 (13–21)	16 (15–23)	0.834	0.9 (0.8–1.1)	0.356	23 (18–32)	20 (16–25)	24 (19–33)			0.182 ^a^		0.95 (0.86–1.05)		0.300
Accelerations, n	5 (3–10)	5 (3–15)	5 (3–10)	0.673	1.02 (0.91–1.14)	0.782	3 (0–5)	3 (1–11)	3 (0–5)			0.588 ^a^		1.1 (0.9–1.3)		0.353
Decelerations, n	11 (8–14)	**14 (11–18)**	**6 (8–14)**	0.007*	**1.22** **(1.05–1.42)**	0.011*	3 (0–7)	4 (0–12)	3 (1–6)			0.893 ^a^		1.1 (0.9–1.3)		0.533
Variable																
Uncomplicated	10 (7–14)	**12 (9–15)**	**9 (7–13)**	0.033*	**1.25** **(1.05–1.49)**	0.012*	3 (0–6)	3 (0–10)	3 (1–5)			0.919 ^a^		1.1 (0.8–1.4)		0.639
Complicated	0 (0–0)	0 (0–0)	0 (0–0)	-^f^	-^f^	-^f^	0 (0–0)	0 (0–0)	0 (0–0)			-^f^		-^f^		-^f^
Other– non-classifiable	1 (0–2)	2 (0–4)	1 (0–2)	0.343	1.2 (0.8–1.6)	0.406	0 (0–1)	1 (0–2)	0 (0–1)			0.375 ^a^		1.4 (0.6–3.2)		0.391
Late	4 (3–6)	5 (3–10)	4 (3–6)	0.121	1.20 (0.96–1.49)	0.110	1 (0–3)	2 (0–4)	1 (0–2)			0.945 ^a^		1.1 (0.7–1.6)		0.782
Uterine contractions, n	16 (10–21)	**19 (13–23)**	**13 (9–18)**	0.027*	**1.1** **(1.0-1.2)**	0.049*	3 (0–5)	3 (0–4)	3 (0–5)			0.783^a^		0.9 (0.7–1.2)		0.586

*IQR* Interquartile range, *FHR* Fetal heart rate, *Bpm*  beats per minute, *fHRV* fetal heart rate variability, *OR*  odds ratio, *CI* coincidence interval

*Statistical significance (*p* < 0,05)

^**a**^Measures are presented in the first stage of labour as n/hour, and for the second stage as n/total duration second stage. Only CTG recordings with a continuous duration during the last 60 min of the first stage of labour were included. The median duration of CTG recording during the second stage was 11 min (IQR 2–23)

^**b**^Low Apgar group: Apgar < 7 at 5 min; Reference group: Apgar ≥ 7 at 5 min

^**c**^Mann-Whitney U test, for non-normally continuous data

^**d**^Multiple logistic regression analysis, with *Nullipara* and *Corticosteroids* as confounders

^**e**^Logistic regression analysis, without confounders due to < 10 cases per group

^**f**^No analysis possible due to no cases

Boldface text indicates statistically significant results

In the second stage of labor, median baseline FHR was 143 bpm, median fHRV was 23 bpm, there was a median of three accelerations, three variable decelerations (all classified as uncomplicated variable decelerations), one late deceleration and three uterine contractions (Table 2). No statistically significant differences in CTG characteristics between the group with low Apgar score and the reference group were observed during the second stage of labor (Table 2).

CTG patterns suggestive of slowly evolving hypoxia were observed in all cases during the first stage of labor, of which 53 (96%) showed normal baseline FHR and normal fHRV with decelerations, and two (4%) showed tachycardia with normal fHRV and decelerations. During the second stage, six cases (25%) showed no CTG-signs of slowly evolving hypoxia, seventeen (71%) showed normal baseline and fHRV with decelerations, and one (4%) showed tachycardia with normal fHRV and decelerations. No differences were observed between the group with low Apgar score and the reference group for both stages of labor. Furthermore, no patterns consistent with subacute or acute hypoxia were observed during both stages of labor (Table 3).

**Table 3:** Cardiotocographic (CTG) characteristics in fetuses with low Apgar score compared to fetuses without this outcome, during the last hour of the first stage of labor and during second stage of labor

CTG characteristics (*n*, %)	First stage of labor			Second stage of labor		
	Total (*n* = 55)	Low Apgar score^a^ (*n* = 20)	Reference group^a^ (*n* = 35)	Total (*n* = 24)	Low Apgar score^a^ (*n* = 6)	Reference group^a^ (*n* = 18)
*Slowly evolving hypoxia*						
Normal baseline + no decelerations + normal fHRV (normal)	0 (0%)	0 (0%)	0 (0%)	6 (25%)	2 (33%)	4 (22%)
Normal baseline + decelerations + normal fHRV	53 (96%)	19 (95%)	34 (97%)	17 (71%)	4 (67%)	13 (72%)
Tachycardia + decelerations + normal fHRV	2 (4%)	1 (5%)	1 (3%)	1 (4%)	0 (0%)	1 (6%)
Tachycardia + decelerations + reduced fHRV	0 (0%)	0 (0%)	0 (0%)	0 (0%)	0 (0%)	0 (0%)
*Subacute hypoxia*						
No complicated variable decelerations (normal)	55 (100%)	20 (100%)	35 (100%)	24 (100%)	6 (100%)	18 (100%
Complicated variable decelerations with normal fHRV	0 (0%)	0 (0%)	0 (0%)	0 (0%)	0 (0%)	0 (0%)
Complicated variable decelerations with reduced fHRV	0 (0%)	0 (0%)	0 (0%)	0 (0%)	0 (0%)	0 (0%)
*Acute hypoxia*						
Baseline FHR > 80 bpm and/or normal fHRV (normal)	55 (100%)	20 (100%)	35 (100%)	24 (100%)	6 (100%)	18 (100%)
Baseline FHR < 80 bpm + loss of fHRV	0 (0%)	0 (0%)	0 (0%)	0 (0%)	0 (0%)	0 (0%)

a Low Apgar group: Apgar < 7 at 5 min; Reference group: Apgar ≥ 7 at 5 min

*fHRV* fetal heart rate variability, *bpm* beats per minute, *FHR *fetal heart rate

In most of the neonates, arterial umbilical cord blood gas analysis was available (*n* = 51). Only one neonate demonstrated biochemical evidence of asphyxia, and this neonate belonged to the group with a low 5‑minute Apgar score. Due to the very low incidence of biochemical asphyxia, no statistical analyses were performed.

Regarding the secondary composite outcome of perinatal mortality and/or severe neonatal morbidity, no statistically significant differences in CTG characteristics were observed between the low Apgar group and the reference group. Given the extensive volume of output, a detailed breakdown of these outcomes is presented in Supplementary Tables S1–S4 for completeness.

## Discussion

This explorative study provides a descriptive overview of intrapartum CTG characteristics in extremely preterm births (24–28 weeks gestation), a group for whom evidence-based interpretation frameworks are lacking. Despite neurological and autonomic immaturity, CTG tracings frequently displayed recognizable features, including baseline FHR, fHRV and both accelerative and decelerative responses. The results of this study underline previous descriptions of CTG characteristics in extremely preterm fetuses [23]. Notably, CTG traces of fetuses with low 5-minute Apgar score exhibited significantly more decelerations, particularly uncomplicated variable decelerations, and uterine contractions than those without. While CTG patterns classically associated with fetal hypoxia at term were rarely seen, the increased frequency of decelerations and contractions may indicate heightened intrapartum stress [36]. However, this should not be interpreted as a definitive marker of stress; given the physiological immaturity and limited normative data at this gestational age, their predictive value remains uncertain.

This study has several strengths and limitations. First, the use of computerized CTG assessment ensured systematic, consistent, and reproducible analysis, addressing the well-documented intra- and interobserver variability associated with manual interpretation [9]. Nevertheless, its retrospective and descriptive design restricts causal inference and firm conclusions. The small sample size and varying (short) duration of the second stage of labor may limit generalizability. However, these limitations are inherent to the rarity of such cases and the inclusion criteria required for this analysis. Despite the limited sample, the number of cases per group was sufficient to allow adjustment for statistically significant confounders in the logistic regression model for the first stage of labor. However, the small number of outcome events inherently restricted the number of covariates that could be included, which represents a limitation of this study. As a result, only a minimal set of confounders could be adjusted for, and important residual confounding cannot be excluded. This limitation may affect the independence of the observed associations and must be taken into account when interpreting the results. Therefore, findings should be regarded as exploratory, and confirmation in larger multicentre cohorts is required before drawing firm clinical conclusion. Another limitation is, that although further gestational‑age–specific analyses in 2‑week intervals would be of physiological interest, the sample size in this exploratory cohort is insufficient to support meaningful subgrouping without substantial loss of statistical validity. Therefore larger multicentre datasets will be required to investigate developmental trajectories of CTG characteristics across finer gestational age windows. Furthermore, CTG interpretation was based on classification systems developed for term fetuses, which may not fully apply to extremely preterm fetuses. To address this, classification was grounded in pathophysiological explanatory models [1, 4, 23]. For deceleration and accelerations definitions, we used the 15 × 15 rule (an amplitude of ≥ 15 bpm lasting ≥ 15 s), rather than the 10 × 10 (≥ 10 bpm for ≥ 10 s), proposed by some for extremely preterm fetuses [23]. In the absence of conclusive evidence favoring one over the other, we adopted the 15 × 15 rule, which is currently endorsed by clinical guidelines [1]. 

Because this study was exploratory and the sample size limited, we deliberately did not perform parallel analyses using unvalidated alternative thresholds such as the 10 × 10 rule, as this would have fragmented the results and reduced interpretability without providing evidence‑based additional insight. Signal loss was a challenge, particularly at earlier gestational ages, and exclusion of cases with > 33% signal loss could have introduced bias.

Although our sensitivity analysis did not show differences in neonatal outcomes between included and excluded cases, the exclusion of CTGs with > 33% signal loss may still bias the cohort toward labors with more stable monitoring conditions, and thus may limit generalizability to fetuses experiencing more severe compromise or rapidly evolving intrapartum deterioration. The choice of primary outcome (low 5-minute Apgar score) requires consideration. The Apgar score alone has been debated as a reliable marker of asphyxia, with some arguing it reflects overall condition rather than specific pathophysiology [37, 38]. However, recent robust evidence supports the prognostic value of the 5-minute Apgar score, even in very preterm infants [33, 39]. A large multinational cohort study found that a 5-minute Apgar < 7 is strongly associated with increased risks of mortality and severe morbidity before 32 weeks’ gestation [33]. These findings reaffirm data showing the Apgar score to be a better predictor of neonatal death than umbilical cord pH alone, particularly in compromised preterm infants [40]. Moreover, in clinical practice, Apgar scores are almost always available — even in emergencies or when blood sampling is not feasible. This makes it a more consistently available and less selectively missing outcome measure than umbilical cord blood gas values, which are often missing in compromised or extremely preterm neonates. This pragmatic consideration supports its use in population-based research. Our findings are consistent with literature highlighting the Apgar score’s continued relevance when used appropriately and interpreted within the clinical context [41].

Also, even though arterial umbilical cord blood gases were available for most neonates in this study, only one case fulfilled biochemical criteria for asphyxia, limiting the ability to draw conclusions about associations between CTG features and acid–base status. This, together with the lack of association with the composite neonatal outcome, underscores the exploratory nature of our findings and highlights the need for gestational‑age–specific CTG frameworks that better correspond to early neonatal physiology. The absence of differences in severe neonatal morbidity and mortality between infants with low versus normal Apgar scores also warrants consideration. Although low Apgar scores are strongly associated with adverse outcomes in very preterm populations, our sample size was limited, reducing the statistical power to detect differences in relatively infrequent endpoints. In addition, modern neonatal resuscitation and intensive care may mitigate the progression from initial neonatal depression to severe short‑term morbidity, thereby attenuating the observable association between early clinical condition and early morbidity in small cohorts. Together, these factors likely explain why the prognostic relationship described in large population‑based studies could not be reproduced in this exploratory dataset. Finally, although CTG characteristics showed no statistically significant associations, decelerations were significantly more frequent in neonates with low 5-minute Apgar score, suggesting that some CTG features may have predictive value. This supports the relevance of intrapartum monitoring of extremely preterm fetuses and highlights the need for larger studies to confirm these findings. To the best of our knowledge, this is the first study to systematically describe CTG characteristics and pathophysiological patterns during extremely preterm labor in relation to perinatal outcomes, providing a valuable foundation for future research.

Few studies have systematically described CTG characteristics in extremely preterm infants in relation to perinatal outcomes. Prior research suggests that the absence of variability or accelerations may be less indicative of fetal compromise before 28 weeks gestation, due to immature autonomic nervous system [23]. Yet, our findings - showing baseline variability and occasional accelerations - suggest a degree of autonomic responsiveness even near the threshold of viability. Variable decelerations were frequently observed in our cohort, which is consistent with findings in term pregnancies where these are often benign and related to cord compression and baroreceptor reflexes. In extremely preterm fetuses, however, the physiological mechanisms underlying decelerative patterns differ substantially. The higher frequency of uncomplicated variable decelerations observed in the low‑Apgar group is unlikely to reflect classic hypoxic processes as described at term. Instead, these associations may indicate limited fetal reserve, immature autonomic control, or reduced tolerance to mechanical or contraction‑related stress [23]. This supports the notion that term‑based CTG interpretation frameworks are not applicable in extreme prematurity and underscores the need for gestational age–specific interpretative models. Furthermore, these observations are consistent with earlier work by Afors & Chandraharan (2011) [23], who also emphasized that classical CTG abnormalities may have different significance in preterm fetuses, further supporting the need for gestational‑age–specific interpretative frameworks. While no significant differences in late decelerations were observed, the small sample size limits definitive interpretation. The observed trend, however, aligns with prior findings [36] and suggests that some CTG features may still carry clinical relevance at this gestational age.

Although machine‑learning approaches such as classification and regression trees could theoretically support data‑driven pattern identification, the exploratory nature and size of the present cohort did not allow for the reliable application of such methods. No differences were observed in predefined CTG patterns, derived from term fetal physiology, that are typically associated with evolving hypoxia. This absence of hypoxia‑related patterns is most likely explained by the fact that intrapartum hypoxia was nearly absent in this cohort. Only one neonate (2%) met biochemical criteria for asphyxia, meaning that our analysis predominantly reflects CTG features in infants with a low 5‑minute Apgar score rather than in hypoxic fetuses. As a result, the classical subacute or acute hypoxia patterns described at term could not reasonably be expected to occur in this dataset. The finding that these patterns were also not present within the low‑Apgar subgroup suggests that early neonatal depression in extreme prematurity may not be preceded by the same CTG manifestations observed at term, highlighting the need to consider alternative or prematurity‑specific CTG features in future research.

## Conclusion

This study is the first to systematically describe intrapartum CTG characteristics in extremely preterm births in relation to perinatal outcomes. Despite neurological immaturity, CTG traces often showed recognizable features, including baseline, variability, and both accelerative and decelerative responses. Uncomplicated variable decelerations and uterine contractions were more frequent in neonates with low 5-minute Apgar score, suggesting that these features, typically benign in term infants, may reflect pathological processes in extreme prematurity. These findings raise concerns about the adequacy of term-based CTG interpretation frameworks for this vulnerable group. As active management of extremely preterm births becomes more common, there is an urgent need to develop gestational age-specific CTG reference standards and predictive models linking CTG features to neonatal outcomes. Interpretation remains challenging due to the multifactorial nature of perinatal outcomes and limited normative data. Future studies should focus on refining CTG definitions and frameworks tailored to extreme prematurity and on linking CTG characteristics to both short- and long-term neonatal outcomes.

## Supplementary Information

Supplementary Material 1.

## Data Availability

The datasets analysed during the current study are not publicly available due to privacy reasons, but are available from the corresponding author on reasonable request.

## Abbreviations

Bpm: Beats per minute
CCS: Corticosteroids
CS: Cesarean section
CTG: Cardiotocography
FHR: Fetal heart rate
fHRV: Fetal heart rate variability
