# Supplementary material for: Intrapartum cardiotocographic patterns and perinatal outcomes in extremely preterm births: an exploratory retrospective cohort study
Source: BMC Pregnancy Childbirth. 2026 Mar 27;26:494. doi: 10.1186/s12884-026-08954-0 (PMC13147894; doi:10.1186/s12884-026-08954-0)
Supplement: Supplementary file 1 — Supplementary Material 1. [file 12884_2026_8954_MOESM1_ESM.docx]

**S1 Table: Baseline characteristics**

|  | **Total (n=55)** | **Composite outcome^c^ (n=23)** | **No composite outcome^c^ (n=32)** | **P-value** |
| --- | --- | --- | --- | --- |
| **Maternal characteristics** |  |  |  |  |
| Ethnicity (n, %)  Caucasian  Non-Caucasian | 46 (84%) 9 (16%) | 19 (83% 4 (17%) | 27 (84%) 5 (16%) | 1.000^a^ |
| Age (Mean, SD) | 29 (5) | **32 (5)** | **28 (6)** | **0.020*^b^** |
| BMI, pre pregnancy  (Mean, SD) | 26 (5) | 26 (5) | 25 (5) | 0.558^b^ |
| Smoking (n, %)  No smoking  Smoking | 48 (87%) 7 (13%) | 19 (83%) 4 (17%) | 29 (91%) 3 (9%) | 0.435^a^ |
| **Obstetrical characteristics** |  |  |  |  |
| First Gestation(n, %) | 30 (55%) | 12 (52%) | 18 (56%) | 0.765^a^ |
| Primipara (n, %) | 38 (69%) | 15 (65%) | 23 (72%) | 0.598^a^ |
| Gestational Age, in days (Mean, SD) | 184 (8) | **181 (8)** | **187 (7)** | **0.003*^b^** |
| Obstetrical disorder (n, %)  Preeclampsia  Fetal growth restriction  Intra-amniotic infection  Vaginal blood loss   Maternal diabetes | 0 (0%) 3 (6%) 1 (2%) 27 (49%) 3 (6%) | 0 (0%) 1 (4%) 0 (0%) 14 (61%) 1 (4%) | 0 (0%) 2 (6%) 1 (3%) 13 (41%) 2 (6%) | -^d^ 1.000^a^ 1.000^a^ 0.139^a^ 1.000^a^ |
| PPROM (n, %) | 28 (51%) | 11 (48%) | 17 (53%) | 0.698^a^ |
| Medication during or in short period before birth (n, %)   Tocolytics  Corticosteroids  None  <2 days  2-14 days  >14 days  Magnesium sulphate | 50 (91%)  0 (0%) 16 (29%) 35 (64%) 4 (7%) 53 (96%) | 21 (91%)  0 (0%) 8 (35%) 12 (52%) 3 (13%) 22 (96%) | 29 (91%)  0 (0%) 8 (25%) 23 (72%) 1 (3%) 31 (97%) | 1.000^a^ 0.216^a^     1.000^a^ |
| Epidural analgesia (n, %) | 11 (20%) | 3 (13%) | 8 (25%) | 0.326^a^ |
| Caesarean section (n, %) | 8 (15%) | 5 (22%) | 3 (9%) | 0.257^a^ |
| **Child characteristics** |  |  |  |  |
| Male sex (n, %) | 25 (46%) | 11 (48%) | 14 (44%) | 0.765^a^ |

**a** Chi-square test including bootstrapping, for categorical data
**b** Unpaired t-test, for normally continuous data
**c** Composite outcome of perinatal mortality (deaths that occur after a gestational age of ≥22 weeks or, if unknown fetal birthweight >500 gram, until 28 days after birth) and/or severe perinatal morbidity, defined as either intraventricular haemorrhage (IVH) grade three or more, periventricular leukomalacia (PVL) grade two or more, moderate or severe bronchopulmonary dysplasia (BPD), necrotizing enterocolitis (NEC) grade two or more or retinopathy of prematurity (ROP) necessitating laser therapy.
**d** No analysis possible due to no cases
* Statistical significance (p<0,05)

**S2 Table: Cardiotocographic (CTG) characteristics during the last hour of the first stage of labour^◊^**

| **CTG Characteristics  (median, IQR)** ^e^ | **Total (n=55)** | **Composite outcome ^c^ (n =23)** | **No Composite outcome ^c^ (n=32)** | **P-value^a^** | **OR  (95% CI)** | **P-value^b^** |
| --- | --- | --- | --- | --- | --- | --- |
| **Baseline FHR, bpm** | 145  (139-151) | 142  (139-147) | 147  (139-153) | 0.094 | 1.2 (0.9-1.6) | 0.235 |
| **fHRV, bpm** | 18 (15-23) | 19 (15-24) | 17 (14-22) | 0.226 | 0.39 (0.03-5.73) | 0.495 |
| **Accelerations, n/hour** | 5 (3-10) | 5 (3-8) | 7 (3-12) | 0.320 | 0.8 (0.5-1.4) | 0.497 |
| **Decelerations, n/hour** | 11 (8-14) | 11 (8-14) | 12 (8-16) | 0.925 | 1.1  (0.7-1.9) | 0.631 |
| Variable |  |  |  |  |  |  |
| Uncomplicated | 10 (7-14) | 10 (7-13) | 10 (7-14) | 0.945 | 1.1  (0.7-1.9) | 0.631 |
| Complicated | 0 (0-0) | 0 (0-0) | 0 (0-0) | -^d^ | -^d^ | -^d^ |
| Other– non-classifiable | 1 (0-2) | 1 (0-2) | 1 (0-2) | 0.549 | 0.4  (0.0-NA^f^) | 1.000 |
| Late | 4 (3-6) | 3 (3-5) | 5 (3-7) | 0.284 | 0.6  (0.1-3.4) | 0.563 |
| **Uterine contractions**, **n/hour** | 16 (10-21) | 13 (9-18) | 18 (10-22) | 0.112 | 0.9 (0.7-1.2) | 0.590 |

**a** Mann-Whitney U test, for non-normally continuous data
**b** Multiple logistic regression analysis, with *Age in years* and *Gestational Age in days* as confounders
**c** Composite outcome of perinatal mortality (deaths that occur after a gestational age of ≥22 weeks or, if unknown fetal birthweight >500 gram, until 28 days after birth) and/or severe perinatal morbidity, defined as either intraventricular haemorrhage (IVH) grade three or more, periventricular leukomalacia (PVL) grade two or more, moderate or severe bronchopulmonary dysplasia (BPD), necrotizing enterocolitis (NEC) grade two or more or retinopathy of prematurity (ROP) necessitating laser therapy.
**d** No analysis possible due to no cases
**e** Only CTG recordings with a continuous duration during the last 60 minutes of the first stage of labour were included.
**f** The upper limit could not be estimated because of model instability
* Statistical significance (p<0,05)
IQR= interquartile range, FHR= fetal heart rate, bpm = beats per minute, fHRV= fetal heart rate variability, NA= Non-applicable, OR = odds ratio, CI= confidence interval

**S3 Table: Cardiotocographic (CTG) characteristics during the second stage of labour^◊^**

| **CTG Characteristics  (Median, IQR)** ^e^ | **Total (n=24)** | **Composite outcome ^c^ (n =8)** | **No Composite outcome ^c^ (n=16)** | **P-value^a^** | **OR  (95% CI)** | **P-value^b^** |
| --- | --- | --- | --- | --- | --- | --- |
| **Baseline FHR, bpm** | 143  (137-148) | 142  (138-148) | 143  (136-148) | 0.713 | 1.0  (0.9-1.1) | 0.915 |
| **fHRV, bpm** | 23 (18-32) | 22 (10-30) | 24 (18-32) | 0.298 | 0.95 (0.87-1.04) | 0.253 |
| **Accelerations, n/total duration** | 3 (0-5) | 2 (0-4) | 4 (0-5) | 0.534 | 0.9 (0.7-1.1) | 0.297 |
| **Decelerations, n/total duration** | 3 (0-7) | 2 (0-6) | 4 (1-8) | 0.264 | 0.9 (0.7-1.1) | 0.307 |
| Variable |  |  |  |  |  |  |
| Uncomplicated | 3 (0-6) | 2 (0-5) | 4 (1-8) | 0.321 | 0.9 (0.7-1.2) | 0.417 |
| Complicated | 0 (0-0) | 0 (0-0) | 0 (0-0) | -^d^ | -^d^ | -^d^ |
| Other– non-classifiable | 0 (0-1) | 0 (0-1) | 0 (0-2) | 0.257 | 0.4 (0.1-1.7) | 0.220 |
| Late | 1 (0-3) | 1 (0-2) | 1 (0-4) | 0.282 | 0.7 (0.4-1.2) | 0.223 |
| **Uterine contractions**, **n/total duration** | 3 (0-5) | 3 (0-5) | 3 (0-6) | 0.900 | 0.95  (0.77-1.18) | 0.663 |

**a** Mann-Whitney U test, for non-normally continuous data
**b** Logistic regression analysis (without confounders due to <10 cases per group)
**c** Composite outcome of perinatal mortality (deaths that occur after a gestational age of ≥22 weeks or, if unknown >500 gram, until 28 days after birth) and/or severe perinatal morbidity, defined as either intraventricular haemorrhage (IVH) grade three or more, periventricular leukomalacia (PVL) grade two or more, moderate or severe bronchopulmonary dysplasia (BPD), necrotizing enterocolitis (NEC) grade two or more or retinopathy of prematurity (ROP) necessitating laser therapy.
**d** No analysis possible due to no cases
**e** The median duration of CTG recording during the second stage was 11 minutes (IQR 2-23)
* Statistical significance (p<0,05)
IQR= interquartile range, FHR= fetal heart rate, bpm = beats per minute, fHRV= fetal heart rate variability, OR = odds ratio, CI= confidence interval

**S4 Table: Cardiotocographic (CTG) characteristics in foetuses with composite outcome compared to foetuses without composite outcome, during the last hour of the first stage of labour and during second stage of labour.**

| **CTG characteristics**  **(n, %)** | **First stage of labour** | | | | **Second stage of labour** | | |
| --- | --- | --- | --- | --- | --- | --- | --- |
|  | **Total  (n=55)** | **Composite outcome^a^ (n=23)** | **No Composite outcome^a^ (n=32)** | **Total  (n=24)** | | **Composite outcome^a^ (n=8)** | **No Composite outcome^a^ (n=16)** |
| *Slowly evolving hypoxia* |  |  |  |  | |  |  |
| Normal baseline + no decelerations + normal fHRV (normal) | 0 (0%) | 0 (0%) | 0 (0%) | 6 (25%) | | 3 (38%) | 3 (19%) |
| Normal baseline + decelerations + normal fHRV | 53 (96%) | 23 (100%) | 30 (94%) | 17 (71%) | | 5 (63%) | 12 (75%) |
| Tachycardia + decelerations + normal fHRV | 2 (4%) | 0 (0%) | 2 (6%) | 1 (4%) | | 0 (0%) | 1 (6%) |
| Tachycardia + decelerations + reduced fHRV | 0 (0%) | 0 (0%) | 0 (0%) | 0 (0%) | | 0 (0%) | 0 (0%) |
|  |  |  |  |  | |  |  |
| *Subacute hypoxia* |  |  |  |  | |  |  |
| No complicated variable decelerations (normal) | 55 (100%) | 23 (100%) | 32 (100%) | 24 (100%) | | 6 (100%) | 18 (100%) |
| Complicated variable decelerations with normal fHRV | 0 (0%) | 0 (0%) | 0 (0%) | 0 (0%) | | 0 (0%) | 0 (0%) |
| Complicated variable decelerations with reduced fHRV | 0 (0%) | 0 (0%) | 0 (0%) | 0 (0%) | | 0 (0%) | 0 (0%) |
|  |  |  |  |  | |  |  |
| *Acute hypoxia* |  |  |  |  | |  |  |
| Baseline FHR >80 bpm and/or normal fHRV (normal) | 55 (100%) | 23 (100%) | 32 (100%) | 24 (100%) | | 6 (100%) | 18 (100% |
| Baseline FHR <80 bpm + loss of fHRV | 0 (0%) | 0 (0%) | 0 (0%) | 0 (0%) | | 0 (0%) | 0 (0%) |

**a** Composite outcome is defined as perinatal mortality (deaths that occur after a gestational age of ≥22 weeks or, if unknown > 500 gram, until 28 days after birth, and/or severe perinatal morbidity, defined as either intraventricular haemorrhage (IVH) grade three or more, periventricular leukomalacia (PVL) grade two or more, moderate or severe bronchopulmonary dysplasia (BPD), necrotizing enterocolitis (NEC) grade two or more or retinopathy of prematurity (ROP) necessitating laser therapy.
fHRV= fetal heart rate variability, bpm= beats per minute, FHR= fetal heart rate
